# Inductive Paper-Based Flexible Contact Force Sensor Utilizing Natural Micro-Nanostructures of Paper: Simplicity, Economy, and Eco-Friendliness

**DOI:** 10.3390/mi15070890

**Published:** 2024-07-07

**Authors:** Haozhe Zhang, Junwen Zhu, Yujia Yang, Qiang Liu, Wei Xiong, Xing Yang

**Affiliations:** 1 Department of Precision Instrument, Tsinghua University, Beijing 100084, China; zhanghz18@tsinghua.org.cn (H.Z.); zhujw21@mails.tsinghua.edu.cn (J.Z.); yang-yj23@mails.tsinghua.edu.cn (Y.Y.); qiangliu@tsinghua.edu.cn (Q.L.); 2 Key Laboratory of Photonic Control Technology (Tsinghua University), Ministry of Education, Beijing 100084, China; 3 State Key Laboratory of Precision Space-Time Information Sensing Technology, Beijing 100084, China; 4 Key Laboratory of Advanced Reactor Engineering and Safety of Ministry of Education, Collaborative Innovation Center of Advanced Nuclear Energy Technology, Institute of Nuclear and New Energy Technology, Tsinghua University, Beijing 100084, China

**Keywords:** flexible sensor, inductive sensor, micro–nanostructure, contact force sensor, paper-based sensor

## Abstract

Inductive contact force sensors, known for their high precision and anti-interference capabilities, hold significant potential applications in fields such as wearable and medical monitoring devices. Most of the current research on inductive contact force sensors employed novel nanomaterials as sensitive elements to enhance their sensitivity and other performance characteristics. However, sensors developed through such methods typically involve complex preparation processes, high costs, and difficulty in biodegradation, which limit their further development. This article introduces a new flexible inductive contact force sensor using paper as a sensitive element. Paper inherently possesses micro- and nanostructures on its surface and interior, enabling it to sensitively convert changes in contact force into changes in displacement, making it suitable for use as the sensor’s sensitive element. Additionally, the advantages of paper also include its great flexibility, low cost, wide availability, and biodegradability. Performance testing on this flexible sensor showed good repeatability, hysteresis, sensitivity, and consistency. When used in experiments for monitoring human motion and respiration, this sensor also exhibited great detection performance. The proposed inductive paper-based flexible contact force sensor, with its simple structure, easy manufacturing process, cost-effectiveness, eco-friendliness, and good sensing performance, provides new insights into research for contact force sensors.

## 1. Introduction

Inductive contact force sensors have the advantages of high accuracy, low power consumption, and strong anti-interference ability, and they hold significant potential applications in fields such as wearable devices, robotics, human–machine interactions, and healthcare [1,2,3,4,5,6,7]. Current research has made progress in improving the sensitivity of inductive contact force sensors. However, there is limited research focusing on the simplicity of the fabrication process, cost, environmental sustainability, and wearing comfort, posing challenges to their widespread practical application. These challenges manifest in two main areas: (i) The sensitive element, as the most critical component of inductive contact force sensor, plays a vital role in enhancing the sensitivity of the sensor [8,9]. Currently, novel nanomaterials such as carbon nanotubes, graphene, and metal nanowires are commonly used for this purpose [10,11,12,13]. While these materials can significantly improve the sensor’s sensitivity, they are generally costly and require complex fabrication processes. This complexity makes it difficult to balance good sensing performance with simple fabrication and low cost, thus limiting the large-scale application of inductive contact force sensors. (ii) The excellent flexibility of flexible substrates aids in conforming inductive contact force sensors to surfaces with various shapes and sizes. However, there is limited research on incorporating flexible substrates into inductive contact force sensors, and current flexible substrates largely consist of polymer materials (e.g., PDMS, PI, PET, and PEN) [14,15,16,17], which are typically difficult to biodegrade and can contribute to environmental pollution. Additionally, integrating flexible substrates with sensitive elements made from novel nanomaterials adds complexity to the sensor fabrication process.

In view of the challenges currently faced by inductive contact force sensors, such as complex fabrication processes, high costs, difficulty in biodegradation, and poor wearing comfort, the following question is raised: is there a material that not only offers advantages in terms of a simple fabrication process, low cost, environmental sustainability, and flexibility, but also provides good sensing performance? This study focuses on paper material, a material extensively used in everyday life, which is known for its low cost, wide availability, and biodegradability [18]. In addition, paper possesses good conformability, making it suitable as a flexible substrate for sensors. More importantly, both the surface and interior of paper contain natural micro-nanostructures, such as micro- and nanofibers. When two sheets of paper are pressed together, the staggered fibers will further form undulating micro–nanostructures at the contact interface, and there are also obvious natural micro–nano porous structures inside the paper. These undulating micro–nanostructures and internal porous structures are compressed under external forces, leading to a macroscopic reduction in paper thickness. This change sensitively converts mechanical variations into displacement changes, thus enabling the paper to effectively act as the sensitive element in contact force sensors. Moreover, paper can act as both the sensitive element and the flexible substrate of contact force sensors, eliminating the need for additional processes to integrate them and thus simplifying the sensor fabrication process to some extent. 

This study exploited the natural micro–nanostructures of paper, simultaneously utilizing it as a sensitive element and flexible substrate for an inductive contact force sensor. By incorporating a layer of copper foil to form an inductive coil and conductor layer, the authors of this study fabricated an inductive paper-based flexible contact force sensor that balances affordability, manufacturing simplicity, and environmental sustainability with suitable performance. Performance testing was conducted on the sensor, and the results indicate that the sensor possesses good repeatability, hysteresis, sensitivity, and consistency. Furthermore, the sensor demonstrated effective performance in experiments involving human motion and respiratory monitoring.

## 2. Device Fabrication

**Figure 1:**
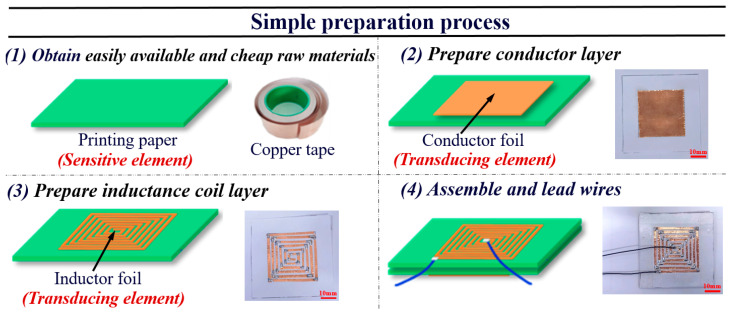
The simple preparation process and actual images of the inductive paper-based flexible contact force sensor.

The fabrication process of this inductive paper-based flexible contact force sensor is depicted in Figure 1, consisting of three main parts: a sensitive element, flexible substrate, and transducing element. Paper serves dual functions as both the flexible substrate and the sensitive element of the sensor, converting the contact force input into a displacement measurement. A layer of copper foil acts as the inductive coil and conductor layer, converting the displacement into an electrical output. The specific fabrication process includes the following steps: Initially, a conductor layer is prepared by cutting a 30 mm × 30 mm piece of copper foil tape (65 μm thickness) and adhering it to a 45 mm × 45 mm sheet of printing paper (No. 7362, Deli, Ningbo, China). Subsequently, the inductive coil layer is prepared on another piece of printing paper of the same size, and both the inductive coil layer and the conductor layer are matched in size (30 mm × 30 mm) and accurately aligned. In actual operation, pasting copper foil (see step 3) is time-consuming, so a mold with a specific shape can be prepared to save time during mass production. It is also worth noting that the dimensions provided here merely serve as an example; the size of the sensor and parameters of the inductive coil (e.g., the turn number, shape, width, etc.) can be adjusted according to specific application needs. Finally, both layers are bonded and sealed using BOPP tape (No. 30325, Deli, Ningbo, China) to fix their relative positions. Leads are attached at the beginning and the end of the inductive coil layer to obtain the sensor’s inductive output signal. Through the above simple preparation process, the inductive paper-based flexible contact force sensor can be fabricated without complex technology or expensive sensitive elements. Instead, commonly available materials (including biodegradable paper and economical and easy-to-process copper foil tape) and simple tools are utilized, resulting in a straightforward, cost-effective, and green preparation process. Additionally, since the leads used for obtaining the electrical signals are positioned on the inductive coil layer but not on the conductor layer, the conductor layer can maintain a smoother surface, enhancing its conformity to human skin and improving wearing comfort.

## 3. Results and Discussion

### 3.1. Sensing Principle and Micro-Nanostructure Characterization

**Figure 2:**
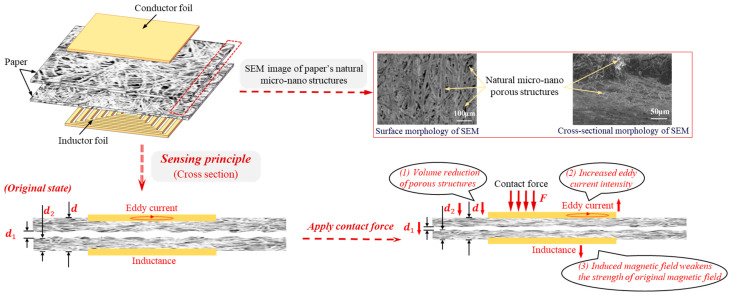
A schematic diagram of the sensing principle of the inductive paper-based flexible contact force sensor along with the SEM characterization of the paper’s surface and cross section.

The sensing principle of the inductive paper-based flexible contact force sensor is illustrated in Figure 2. As described in the fabrication process, the structure of this sensor can be considered as two closely adhered sheets of printing paper, one with a conductor layer and the other with an inductive coil layer. The paper with the conductor layer serves as the force-receiving end of the sensor, while the paper with the inductive coil layer acts as the fixed end. The sensitive element of the sensor is the paper itself. The characterization of the surface and cross-sectional morphology of the printing paper determined using a high-resolution scanning electron microscope (Model 450, NOVA NANOSEM, New York, NY, USA) reveal that the paper contains abundant natural micro-nanostructures composed of a dense arrangement of micro- and nanofibers. The specific sensing principle is as follows: When the two sheets of paper are tightly pressed together, the staggered fibers further form undulating micro–nanostructures at the contact interface. And when contact force is applied to the paper, these structures at the interface and within the paper are compressed, macroscopically resulting in the compression of the sensor. The transducing elements of the sensor are the inductive coil layer and the conductor layer, and its sensing principle is introduced as follows: when an alternating current passes through the inductive coil, an induced electromotive force (EMF) is generated in the conductor layer according to Faraday’s law of electromagnetic induction, resulting in the production of eddy currents. The induced EMF in the conductor layer can be calculated using the following formula:

(1) $$E_{1}=-\frac{d\Phi_{1}}{dt}$$

where $\frac{d\Phi_{1}}{dt}$ is the rate of change in magnetic flux through the conductor layer, and the negative sign indicates that the direction of the induced EMF is opposite to the direction of the change in the magnetic field according to Lenz’s Law. When contact force F is applied, the gaps $d_{1}$ formed by the undulating micro–nanostructures at the interface and the gap $d_{2}$ within the micro–nano porous structure inside the paper both decrease. These changes reduce the overall distance d between the conductor and inductive coil layers. As the magnetic field strength increases with the decrease in the distance d, the magnetic flux $\Phi_{1}$ through the conductor layer also increases. According to Equation (1), $\frac{d\Phi_{1}}{dt}$ increases, thereby increasing the induced EMF $E_{1}$ and the eddy currents, which, in turn, enhance the strength of the induced magnetic field opposing the direction of the inductive coil’s magnetic field. The self-inductance of the inductive coil is calculated sing the following formula:

(2) $$L_{0}=\frac{\Phi_{0}}{I_{0}}$$

where $\Phi_{0}$ is the effective magnetic flux through the inductive coil. As the opposing magnetic field generated by the conductor layer intensifies, the effective magnetic flux $\Phi_{0}$ passing through the inductive coil decreases. According to Equation (2), this results in a decrease in the self-inductance $L_{0}$ of the coil. In summary, as the contact force F increases, the inductance value of the sensor’s inductive coil layer decreases, thus achieving good sensing performance.

### 3.2. Sensor Performance Testing

**Figure 3:**
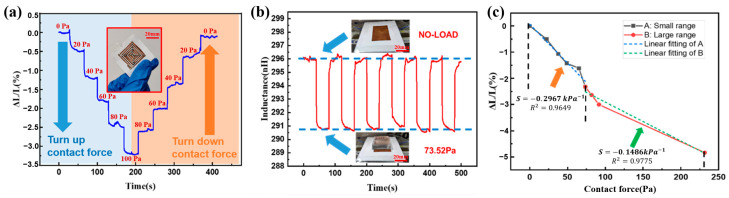
Performance testing of the inductive paper-based flexible contact force sensor. (**a**) The hysteresis curves of the sensor during increasing and decreasing contact forces. (**b**) The repeatability curves of the sensor under multiple applications of the same contact force. (**c**) The sensitivity curves of the sensor in different contact force ranges.

A performance evaluation of the inductive paper-based flexible contact force sensor is conducted, as shown in Figure 3. Here, PDMS blocks with different weights are used as the source of contact force. Using PDMS blocks as the source of contact force pressure instead of a weight or a push–pull force gauge can effectively avoid interference factors, such as conductivity and parasitic capacitance, thereby ensuring the accuracy of the tests. Each PDMS block, cut to the size of 20 × 20 mm^2^ and different heights, are placed on the sensor’s conductor layer to exert a downward force perpendicular to this layer. The sensor’s inductance value is measured using an impedance analyzer (Model WK6500B, Wayne Kerr, Bognor Regis, UK) with an applied AC voltage amplitude of 1 V and a frequency of ~30 MHz.

PDMS blocks of various weights are sequentially loaded onto and then removed from the sensor. As demonstrated in Figure 3a, the sensor’s inductance value progressively decreases as the contact force gradually increases and increases again following the decrease in the force, with minimal hysteresis being observed during the loading and unloading cycles. Figure 3b shows that when the same contact force is repeatedly applied, the variation in the sensor’s inductance remains consistent, indicating good repeatability. The quantitative sensor sensitivity S, illustrated in Figure 3c, is tested using the following formula [19]:

(3) $$S=\frac{∆L/L}{∆P}$$

where ∆L/L represents the rate of change in the sensor output inductance, and ∆P is the magnitude of the contact force applied. The result shows that the sensor exhibits a sensitivity of $S=0.2967{\;kPa}^{-1}$ at a lower contact force range (0~75 Pa) and $S=0.1486\;{kPa}^{-1}$ at a higher contact force range (75~250 Pa). In practical applications, the sensitivity value can be chosen based on the range of the contact force to be measured. The above test results demonstrate that despite its simple fabrication process, low cost, and environmental friendliness, the sensor still performs with good hysteresis, repeatability, and sensitivity.

### 3.3. Sensor Performance Optimization

**Figure 4:**
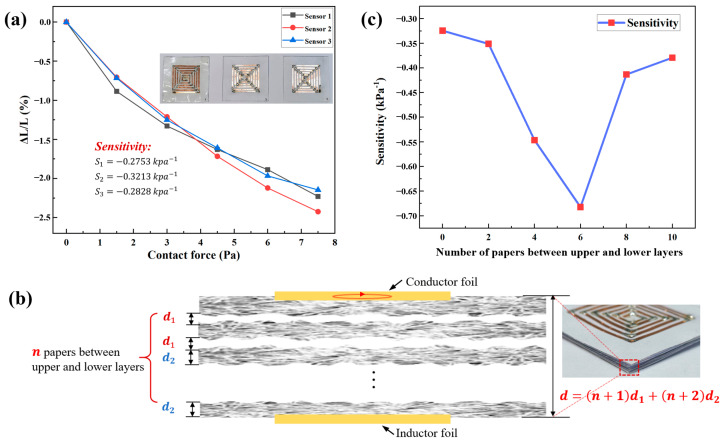
(**a**) The consistency curves of the three sensors made with same preparation process, showing similar sensitivity values. (**b**) A schematic diagram and physical image depicting the increase in the number of papers between the upper and lower layers to increase the $d_{1}$ and $d_{2}$ values. (**c**) The relationship of the sensor’s sensitivity with the number of papers between the upper and lower layers.

We test the consistency of multiple inductive paper-based flexible contact force sensors, as shown in Figure 4a. Using the preparation process described in Figure 1, we fabricate three identical sensors and test their sensitivities in the range of 0~7.5 Pa. The results indicate that the three sensors exhibit similar sensitivity values. This suggests that, despite there being slight variations in the paper surface of different sensors, the micro–nanostructures of the sensors fabricated using the same process are approximately the same in size. Consequently, the batch-to-batch performance variation is minimal, indicating good consistency. We also optimize the performance of the sensor, which operates on the principle that, with an applied contact force, the gaps $d_{1}$ formed by the undulating micro–nanostructures at the interface and the gap $d_{2}$ within the micro–nano porous structure inside the paper both decrease. Therefore, it is intuitive to alter the $d_{1}$ and $d_{2}$ values by increasing the number of papers between the upper and lower layers of the sensor. As shown in Figure 4b, adding n layers of identical paper between the upper and lower layers changes the total thickness of the sensor to $d=\left(n+1\right)d_{1}+(n+2)d_{2}$. We test the sensitivity of the sensors with the addition of 0 (the original state), 2, 4, 6, 8, and 10 layers of paper, as shown in Figure 4c. The results show that as the number of paper layers increases from zero to six, the sensitivity of the sensor gradually increases. This is because with more paper layers, the reduction in total thickness d under the same contact force is greater, leading to a stronger opposing magnetic field to the original magnetic field, and there is a greater decrease in the inductance of the inductive coil, thereby increasing sensitivity. However, as the number of paper layers increases beyond six, the sensitivity of the sensor gradually decreases. This is because the excessive thickness of the paper results in a smaller induced current in the conductor layer, which causes a smaller reduction in the inductance of the coil under the same contact force, thereby leading to decreased sensitivity. Therefore, the sensitivity of the sensor can be improved by appropriately increasing the number of paper layers between the upper and lower layers of the sensor. However, it should be noted that the optimal number of additional paper layers may vary depending on the sensor’s size and the inductive coil’s parameters.

### 3.4. Application of Sensors in Human Motion and Respiratory Monitoring

**Figure 5:**
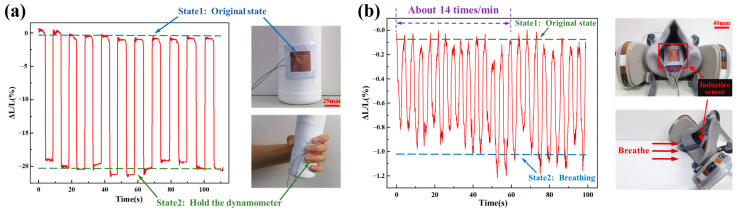
The application of the inductive paper-based flexible contact force sensor for (**a**) measuring handgrip strength and (**b**) monitoring the frequency and intensity of human breathing.

This inductive paper-based flexible contact force sensor is also applied to monitor human movement and respiratory activities. Figure 5a demonstrates the measurement of handgrip strength using this sensor. Due to the sensor’s good flexibility and conformability, it can be adhered to a cylindrical object with a certain curvature, with the conductor layer facing outward, forming a dynamometer model. By repeatedly gripping and then releasing this dynamometer, the inductance value of the sensor varies with the motion, presented as the measured curves, from which the grip strength values can be calculated based on the sensor’s sensitivity. The consistency in grip strength observed in this experiment indicates the sensor’s good repeatability and its ability to achieve accurate handgrip strength measurements. The results also indicate that the paper-based flexible sensor can also be employed to measure other contact forces related to human body motions, such as contact forces at the knee joint, fingers, elbow joint, wrist joint, shoulder joint, etc. Additionally, this sensor is also used to monitor the frequency and intensity of human respiration, as illustrated in Figure 5b. Due to the sensor’s good adhesiveness and thin profile, it can be affixed inside a mask (Model 6001CN, 3M), with the conductor layer facing outward. Since both paper and copper foil are harmless to humans and the environment, wearing the sensor in the long term is permissible. When wearing the mask equipped with the sensor, the airflow generated by breathing applies a contact force to the sensor. The test curve clearly differentiates between two consecutive breaths, demonstrating the sensor’s good sensitivity, and it is sufficient to measure the subtle forces generated by exhalation. The curve also shows that the test participant breathed 14 times in one minute, which falls within the normal adult respiratory rate of 12 to 20 breaths per minute [20], reflecting the accuracy of the sensor measurement. Given that many diseases are characterized by symptoms such as rapid or weak breathing, this paper-based flexible sensor has potential for monitoring respiration during illness or physical activity.

## 4. Conclusions

In summary, this article introduces an inductive paper-based flexible contact force sensor that balances a simple fabrication process, low cost, and environmental sustainability with suitable sensing performance. In principle, through theoretical models and electron microscopy characterization, this study proposes that paper can be used as the sensitive element of an inductive flexible contact force sensor by using the undulating micro–nanostructure formed on the contact surface of two closely adhered sheets of paper and the micro–nano porous structure inside the paper. In terms of sensing performance, the evaluation tests demonstrate that the sensor possesses good hysteresis, repeatability, sensitivity, and consistency. For manufacturing, only printing paper and copper foil tape are used as raw materials, eliminating the need for complicated preparation processes and expensive raw materials while also enhancing environmental sustainability. In terms of application, the sensor shows great performance in experiments involving human motion and respiratory monitoring. Therefore, the inductive paper-based flexible contact force sensor proposed in this article offers new directions for research in contact force sensors and is anticipated to facilitate cost-effective applications in fields such as wearable devices, medical monitoring, and human–computer interaction.

## Data Availability

The data presented in this study are available upon request from the corresponding author.
